# Extracapsular Lymph Node Involvement in Ovarian Carcinoma

**DOI:** 10.3390/cancers11070924

**Published:** 2019-07-01

**Authors:** Sabine Heublein, Heiko Schulz, Frederik Marmé, Martin Angele, Bastian Czogalla, Alexander Burges, Sven Mahner, Doris Mayr, Udo Jeschke, Elisa Schmoeckel

**Affiliations:** 1 Department of Obstetrics and Gynecology, Heidelberg University Hospital, Ruprecht-Karls-University of Heidelberg, 69120 Heidelberg, Germany; 2 Department of Obstetrics and Gynecology, Klinikum der Universität München, Ludwig Maximilians University, 81377 Munich, Germany; 3 Department of Obstetrics and Gynecology, Mannheim University Hospital, 68167 Mannheim, Germany; 4 Department of Surgery, Klinikum der Universität München, Ludwig Maximilians University, 81377 Munich, Germany; 5 German Cancer Consortium (DKTK), 69120 Heidelberg, Germany; 6 Department of Pathology, Ludwig Maximilians University, 81377 Munich, Germany

**Keywords:** ovarian cancer, lymph node metastasis, extra-capsular growth, prognosis

## Abstract

Ovarian cancer (OC) spread to retro-peritoneal lymph nodes is detected in about one out of two patients at primary diagnosis. Whether the histologic pattern of lymph node involvement i.e., intra-(ICG) or extracapsular (ECG) cancer growth may affect patients’ prognosis remains unknown. The aim of the current study was to analyze the prevalence of ECG and ICG in lymph node positive ovarian cancer. We further investigated whether ECG may be related to patients’ prognosis and whether biomarkers expressed in the primary tumor may predict the pattern of lymph node involvement. Lymph node samples stemming from 143 OC patients were examined for presence of ECG. Capsular extravasation was tested for statistical association with clinico-pathological variables. We further tested 27 biomarkers that had been determined in primary tumor tissue for their potential to predict ECG in metastatic lymph nodes. ECG was detected in 35 (24.5%) of 143 lymph node positive patients. High grade (*p* = 0.043), histologic subtype (*p* = 0.006) and high lymph node ratio (LNR) (*p* < 0.001) were positively correlated with presence of ECG. Both ECG (*p* = 0.024) and high LNR (*p* = 0.008) were predictive for shortened overall survival. A four-protein signature determined from the primary tumor tissue was associated with presence of concomitant extracapsular spread in lymph nodes of the respective patient. This work found extracapsular spread of lymph node metastasis to be a common feature of lymph node positive ovarian cancer. Since ECG was positively associated with grade, LNR and shortened overall survival, we hypothesize that the presence of ECG may be interpreted as an indicator of tumor aggressiveness.

## 1. Introduction

Metastatic spread to lymph nodes is common in solid tumors and is counted as one of the most potent prognostic determinants. Apart from lymph node involvement per se, the histological growth pattern of neoplastic cells within the lymph node may add further prognostic information [1,2]. Mainly, the phenomenon of extracapsular growth (ECG), i.e., extension of cancer cell invasion beyond the connective tissue capsule of the lymph node, has been linked to tumor aggressiveness in many cancer entities [1,2,3,4,5,6]. Meanwhile, ECG has become an established prognostic factor for gastrointestinal, breast, and cervical cancer [1,4,5]. It has reproducibly been linked to advanced cancer stage, earlier disease relapse, treatment resistance, and shortened patient survival [1,2,6]. Regarding squamous cell cancer of the vulva and cancers of the head/neck, ECG has even been incorporated into the official cancer staging systems, respectively [7]. 

Though an extensive literature search was performed, we did not find published data on ECG in ovarian cancer (OC). So far, the prevalence of ECG in OC remains elusive. Moreover, it is still unknown whether the presence of ECG might be linked to clinicopathological parameters or prognosis in OC patients.

Therefore, the aim of the current study was to investigate the pattern of lymph node involvement (ECG vs. ICG) in lymph node positive ovarian cancer. We further analyzed whether ECG may be related to FIGO (The International Federation of Gynecology and Obstetrics) stage, histological subtype, tumor grade, residual disease or patients’ prognosis in OC. Finally, we investigated whether a biomarker signature determined from the primary tumor might be associated with ECG in associated lymph nodes.

## 2. Results

### 2.1. Study Cohort

Most patients presented with an extended primary tumor, rated as pT3 (*n* = 125; 87.4%), fewer patients with pT2 (*n* = 12; 8.4%) and pT1 (*n* = 6; 4.2%). According to FIGO 8ed, 127 patients were staged as FIGO III (IIIA: *n* = 18 (12.6%), IIIB: *n* = 17 (11.9%), IIIC: *n* = 92 (64.3%)) while the remaining 16 (11.2%) patients were assigned stage IV disease. Histological characterization revealed serous histologic differentiation in 129 cases (90.2%). Other subtypes were distributed as follows: endometroid (*n* = 5; 3.5%), clear cell (*n* = 4; 2.8%), mucinous (*n* = 1; 0.7%), and undifferentiated (*n* = 4; 2.8%). A percentage of 85.8% (109 out of 129) of serous cases were graded as high grade. Grading of remaining subtypes was G1 in one, G2 in three and G3 in ten patients.

Data on residual disease after primary debulking surgery were available from 124 out of 143 cases. Complete cytoreduction (i.e., no residual disease) was achieved in 83 (66.9%) out of 124 cases. Numbers of resected lymph nodes ranged from 1 to 94, numbers of positive lymph nodes from 1 to 67. Median count of resected lymph nodes was 37.0 and median count of positive nodes was 5.00 nodes per patient, respectively. Median LNR was 0.17 with a minimum of 0.01 and a maximum of 1.00. Patient’s age at primary diagnosis ranged between 33.4 and 87.6 years with a median age of 59.5 years. Median overall survival of the cohort was 45.8 months (95% CI: 32.4–59.3) and median follow up time was 87.6 months (95% CI: 48.5–125.7). There were 80 observed deaths. 

**Figure 1:**
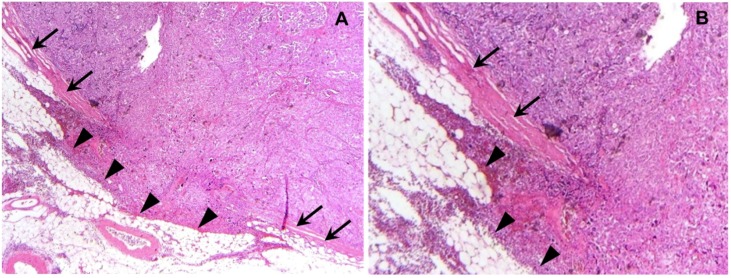
Extracapsular growth of ovarian cancer in a retroperitoneal lymph node: A representative photomicrograph (10× lens (**A**), 20× lens (**B**), HE staining) of extracapsular growth in ovarian cancer is presented. Arrows indicate the intact capsule lining the lymph node that has been invaded by cancer cells. The region of invasive neoplastic cells breaking through the capsule and invading fatty tissue adjacent to the lymph node is indicated by arrowheads (**A**,**B**).

Out of 143 patients analyzed we observed ECG (Figure 1) in 35 (24.5%) and ICG in 108 cases (75.5%). While there was no significant correlation to patient age, tumor size, residual disease, and FIGO stage, ECG was detected more often in cases diagnosed with a high grade than with low grade ovarian primary (*p* = 0.043) (Table 1). Presence of ECG was not related to the number of lymph nodes resected. However, the ratio of positive lymph nodes (LNR) significantly differed depending on the presence of ECG (*p* < 0.001). The proportion of ECG positive cases in patients with a lymph node ratio higher than 0.3 was 22/33 (66.7%) while the presence of ECG in those with lower lymph node ratios was observed in 22 out of 79 patients (27.8%). ECG was more common in those patients whose primary tumor was of non-serous histology (*p* = 0.006). Finally, patient age was not different between ECG and ICG cases.

To rule out potential confounding by different subtypes, analyses were repeated in the serous subtype only. Again, ECG was not correlated with patient age, tumor size, number of total nodes or FIGO stage. While correlation to grading was lost, a lymph node ratio higher than 0.3 was still closely associated with presence of ECG (*p* < 0.001).

### 2.2. Histological Pattern of Lymph Node Involvement Is Associated with the Biomarker Profile of the Primary Tumor 

Immunostaining data for 27 protein markers (Glycodelin and its glyco-modification Glycodelin A, steroid hormone receptors (ERα, ERβ, PRA, PRB, and GPER), gonadotropins-/receptors (hCG, LHCGR, and FSHR), Galectins (Gal-1, Gal-3, Gal-7, Gal-8, and Gal-9), p53, Mucin-1 (as detected by anti-peptide antibodies: VU3C6, VU4H5, HMFG1) and glyco-modifications of Mucin-1 (TA-MUC1 as detected by Gatipotuzumab, TF (CD176)) were available in 32 ovarian cancer cases. Galectins were determined in cancer cell nuclei, cancer cell cytoplasm and/or tumor stroma. 

**Figure 2:**
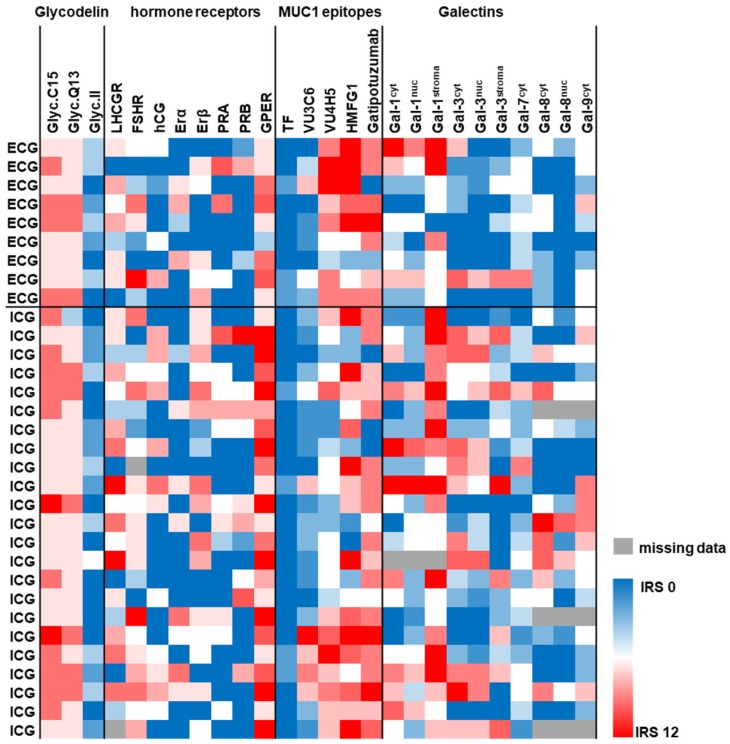
Protein profile of the primary tumor is associated with ECG in associated lymph nodes: Immunostaining data of 27 protein/carbohydrate biomarkers were available from 32 primary tumor tissue cases. IHC data of 26 biomarkers are illustrated as heat map. The protein profile (red—high expression, blue—low expression) of the primary tumor was tested for association with ICG/ECG. IHC was also used to approximate p53 mutational status. Data on p53 in relation to ICG/ECG are presented in the text.

**Figure 3:**
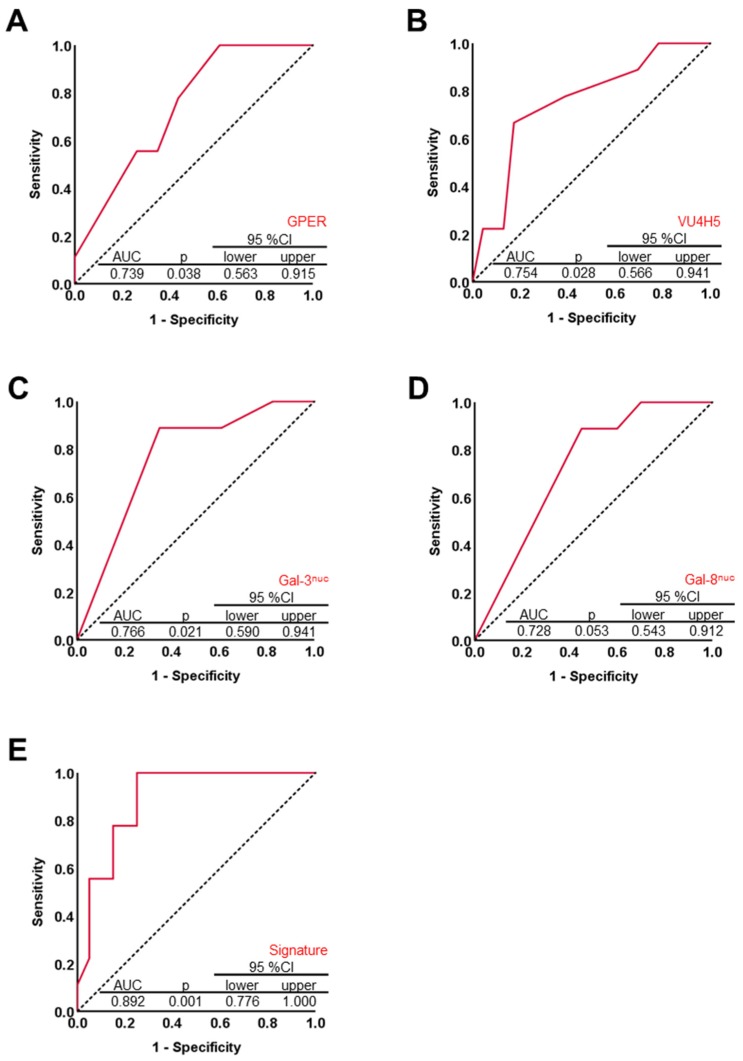
A four-marker signature built of G-Protein coupled estrogen receptor (GPER), MUC-1 and Gal-3/8 is associated with ECG in associated lymph nodes: Four out of 26 markers * turned out to be predictive for presence of ECG in associated retroperitoneal lymph node metastasis (**A**–**D**) as determined by receiver operating characteristic (ROC) analysis using SPSS v22 (**A**–**E**). Diagonal segments are produced by ties. Likelihood of the state event (ECG) was associated with decrease in test variable in case of GPER, Gal-3^nuc^, and Gal-8^nuc^ (**A**,**C**,**D**). Regarding VU4H5, likelihood of the state event (ECG) was associated with increase in test variable (VU4H5, **B**). Primary tumors expressing GPER, Gal-3^nuc^, and Gal-8^nuc^ at low and MUC-1 (as detected by VU4H5) at high levels were more likely to present extracapsular metastatic spread in respective, case-matched lymph node specimens (**A**–**D**). A signature combining GPER, VU4H5, Gal-3, and Gal-8 was calculated and tested for its diagnostic strength to discriminate ECG from ICG cases (**E**). * Data on p53 mutational status (i.e., 27^th^ biomarker) in relation to ICG/ECG are presented in the text. There was no difference regarding the prevalence of ECG and ICG with respect to p53 mutation of the primary tumor.

**Figure 4:**
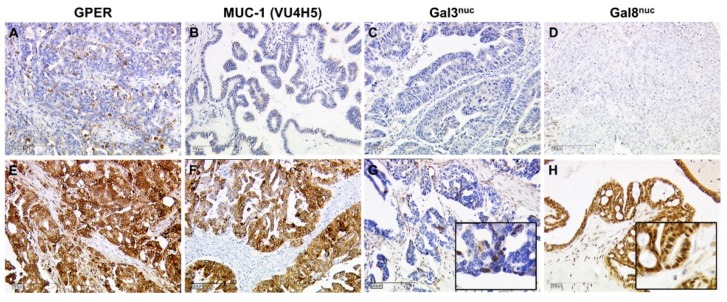
Immunohistochemistry of GPER, MUC-1, and Gal-3n^uc^/8^nuc^ in primary tumor tissue: GPER (**A**,**E**), MUC-1 (**B**,**F**), Gal-3^nuc^ (**C**,**G**), and Gal-8^nuc^ (**D**,**H**) were determined by IHC in primary OC tumor tissue. Representative images of their staining patterns scored as negative (**A**–**D**) and positive (**E**–**H**) are shown. Scale bars represent 200 µm. Inserts in G and H are magnified twice from the original image thus to illustrate nuclear staining of Gal-3 and Gal-8.

Nine out of 32 cases were diagnosed with ECG, while ICG was detected in the remaining 23 cases. Neither Glycodelin nor its immunosupressive glyco-modification Glycodelin A were correlated with the presence of ECG. Out of the steroid hormone receptor set only the G-Protein coupled estrogen receptor (GPER) was associated with the mode of lymph node involvement (*p* = 0.038, Figure 2 and Figure 3A). Median GPER immunopositivity was significantly lower in those primary tumors that presented extracapsular spread in cancer affected lymph nodes (IRS (ICG) = 9.0 vs. IRS (ECG) = 4.0). Though GPER shares some structural similarity with FSHR and LHCGR, neither those two nor HCG, which acts as a ligand on LHCGR, appeared to correlate to the pattern of lymph node involvement. Since there is a strong link between Mucin-1 and metastasis formation, we screened several antibodies detecting either the protein backbone or glyco-modifications of Mucin-1. Only the anti Muc-1 antibody VU4H5 seemed to correlate (*p* = 0.028, Figure 2 and Figure 3B) with ECG in a way that cases that were diagnosed with ECG appeared to overexpress Mucin-1 (IRS (ICG) = 4 vs. IRS (ECG) = 8). Loss of nuclear Galectin-3 (Gal-3) was also predictive (*p* = 0.021) for ECG (Figure 2 and Figure 3C). A similar correlation was found in the case of nuclear Gal-8, though statistically this association was of borderline significance (*p* = 0.053) only (Figure 2 and Figure 3D). Representative photomicrographs of GPER, MUC-1 (VU4H5), Gal-3^nuc^, and Gal-8^nuc^ as detected using immunohistochemistry in primary tumor tissue are shown in Figure 4. A logistic regression was run to ascertain the effects of GPER, MUC-1 (VU4H5), Gal-3^nuc^, and Gal-8^nuc^ on the likelihood of extracapsular spread. The logistic regression model was statistically significant, χ^2^ = 13.29, *p* = 0.01. The model correctly classified 79.3% of cases. To evaluate the fit of the logistic regression model a receiver operating characteristic (ROC) curve was calculated. The combined signature of GPER, MUC-1 (VU4H5), Gal-3^nuc^ and Gal-8^nuc^ proved to be a characteristic of primary tumors presenting with ECG in associated nodes (AUC = 0.892, *p* = 0.001; Figure 3E). The Youden index of our biomarker combination was 0.75. At this cut point sensitivity was 100% and specificity was 75%.

About three-quarters of patients (78.1%, 25/32) were diagnosed with a p53 mutation in their primary tumor—as estimated using the established immunohistochemistry scoring system [8,9]. However, there was no difference regarding the prevalence of ECG and ICG with respect to p53 mutation of the primary tumor.

### 2.3. Evaluation of ECG as a Potential Prognostic Factor for Overall Survival 

**Figure 5:**
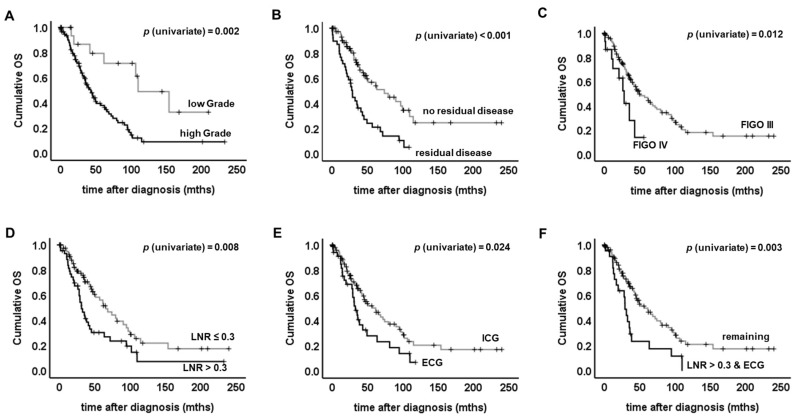
Univariate survival analysis: Log rank tests were performed to determine if there were differences in the survival distribution for the different types of lymph node involvement (ECG vs. ICG), the different fractions of affected lymph nodes (LNR > 0.3 vs. LNR ≤ 0.3) or for clinicopathological parameters. Kaplan–Meier survival curves are shown. High grade (**A**), residual disease after primary debulking surgery (**B**), advanced FIGO stage (**C**) or high LNR (**D**) were found to be associated with shortened overall survival (OS). OS was significantly different when ECG vs. ICG cases were compared (**E**). Long-term surviving patients were only found in within the ICG group (**E**). Those cases presenting both high LNR and ECG at the same time were found to have exceptional reduced OS (**F**).

The number of deaths observed was 25/35 (71.4%) in the ECG group and 55/103 (53.4%) in the ICG group. Median OS in patients diagnosed with ECG was reduced to 32.6 months (95% CI: 25.1–40.0) as compared to 61.2 months in the ICG cohort (95% CI: 39.2–83.2). OS of ECG vs. ICG cases was significantly different and ECG was prognostic for shortened OS (*p* = 0.024, Table 2, Figure 5E). The total number of resected lymph nodes did not influence overall survival (OS), and neither did FIGO stage, tumor size, histologic subtype or patient age (Table 2). Poor differentiation of the primary tumor (i.e., G2/3 in non-serous or high grade in serous cancers), FIGO stage IV and the presence of residual disease were highly prognostic for shortened OS (Grading: *p* = 0.002; FIGO: *p* = 0.012; residual disease: *p* < 0.001; Figure 5A–C, Table 2). An LNR higher than 0.3 (*p* = 0.008) as well as the combination of LNR and ECG (*p* = 0.003) were also associated with shortened OS (Figure 5D,F, Table 2). To exclude potential biasing by clinico-pathological covariates, subgroup analysis was performed. Presence of ECG remained to be prognostic for shortened OS in cases classified as either pT1/2 (*p* = 0.031), FIGO III (*p* = 0.029), or FIGO IV (*p* = 0.030). 

In non-serous cancers the pattern of lymph node involvement (ECG vs. ICG) remained prognostic for shortened OS (*p* = 0.048). Regarding serous cancers, association of ECG and reduced OS did not reach statistical significance (*p* = 0.281), but survival curves were clearly separated three years after surgery, though the difference was not significant (*p* = 0.125). Within all histologic sub-groups long term survivors (i.e., longer than 10 years after surgery) were only found in those cases diagnosed with ICG.

Finally, univariate survival analysis was restricted to those patients with information on residual disease and survival available (*n* = 122) and ECG continued to be associated with shorter OS (*p* = 0.043) in this smaller cohort, too. 

Cox regression (Table S1) was performed to identify independent predictors for OS. Pattern of lymph node involvement (ECG vs. ICG), lymph node ratio and tumor grading failed to remain significant within multivariate testing, while residual disease (*p* = 0.01 and *p* = 0.02) was still predictive in both multivariate testing sets. The second multivariate analysis was run, to test whether the combination of ECG and lymph node ratio may be an independent prognosticator for OS. This combined parameter showed a trend of potentially acting as an independent negative prognosticator (*p* = 0.08) (Table S1).

## 3. Discussion

We herein report that extracapsular spread is a common phenomenon in ovarian cancer. The current study detected extracapsular spread in 24.5% of advanced staged ovarian cancer cases. A literature search revealed a similar prevalence of ECG in different types of cancer e.g., 19.1% in vulvar [10] and 30.1% in cervical [5] cancer patients. We found ECG to be more common in non-serous OC as compared to the serous histological subtype. So far, this observation cannot be explained satisfactorily. However, this finding may further underline tumor biologic differences among different OC subtypes [11]

In general, ECG was closely correlated with tumor grade and a high lymph node ratio, while being independent of resected lymph nodes or tumor size. These observations support the hypothesis that extracapsular spread may be a surrogate marker for tumor biologic features that drive extravasation and thereby cause tumor aggressiveness. We hence questioned whether the protein profile of the ovarian primary may be related to the presence of ECG in associated lymph nodes.

Metastatic spread is majorly determined by tumor biologic features of the primary tumor. In order to conclude on a biomarker profile that may predict extracapsular spread, a set of 27 protein markers (including p53), that had been determined in the ovarian primary tumor, were correlated with presence of ECG. We found that expression of Mucin-1 (as determined by the antibody VU4H5), Gal-3^nuc^, Gal-8^nuc^, and GPER were statistically associated with capsular extravasation. Interestingly, VU4H5—which was positively correlated with ECG in the current study—had been demonstrated to predict reduced overall survival in this ovarian cancer sample [12]. On the contrary, GPER and Gal-3/8 were inversely correlated with presence of ECG and had been found to be positive prognosticators in ovarian cancer [13,14,15]. We introduced a four-marker signature built by VU4H5, GPER, and Gal-3/8 which was predictive for ECG in associated lymph nodes with relatively good sensitivity and specificity. However, in order to validate the robustness of this signature, an independent validation set as well as testing for interactions would be required. It would also be of interest, if this four-marker signature would predict lymph node involvement as such. If so, such a marker set could help to identify a subgroup of patients with high risk of extracapsular nodal involvement, high extent of nodal involvement and hence a considerably poor prognosis. Whether this subgroup of patients may potentially benefit from systematic lymphadenectomy, would have to be addressed in a prospective trial. 

It is well known that complete cytoreduction, FIGO stage and histopathologic subtype are the strongest prognostic factors in epithelial ovarian cancer [16,17]. However, the role of systematic lymphadenectomy within optimal debulking surgery in ovarian cancer has been controversial for years. Recently, results from a large multi-center, prospectively randomized trial of lymphadenectomy in clinically node negative advanced ovarian cancer patients were presented (LION Trial, AGO-OVAR OP.3) [18]. Harter et al. highlighted that omitting lymph node dissection in clinically node negative advanced ovarian cancer patients does not affect prognosis [18]. This has changed the standard of care in many institutions around the world. On the other hand, an earlier, however retrospective exploratory analysis of three prospectively designed randomized chemotherapy trials found that lymphadenectomy was associated with extended overall survival in patients diagnosed with advanced disease that had been optimally debulked [19]. In addition, systematic lymphadenectomy was superior as compared to the removal of bulky nodes only in terms of progression free survival, though it did not affect OS in patients that had undergone optimal tumor debulking [20]. 

These data seem to be partly contradictory at first glance. To some extent this may be due to the retrospective character of earlier studies, different trial designs and various primary endpoints of the studies cited above. Another interpretation may be the existence of patient subgroups that benefit from lymph node dissection to different extents. Such subgroups—if they exist—have not been identified so far. Biomarkers or clinical predictors that aid identifying those patients that benefit most from systematic lymph node removal remain to be defined. Whether the biomarker signature introduced above may help to select OC cases with ECG and presumably highly aggressive disease can just be speculated by now. 

Nowadays, such patients diagnosed as lymph node positive or Stage IIIB or higher may be eligible for a Bevacizumab therapy based on the GOG 218 and ICON 7 trials [21,22]. As a consequence, though potentially missing direct benefit from surgical resection per se, systematic lymph node dissection may aid to perform more accurate staging thereby avoiding adjuvant over- or undertreatment. Whether pattern/extent of lymph node involvement (ECG vs. ICG) may add additional prognostic information in ovarian cancer macroscopically confined to the pelvis may be worth addressing in the future. 

Finally, there are several limitations to our study that need to be critically discussed. In general, further studies and larger patient cohorts are needed in order to validate our findings. Future study setups should also rule out potential confounders like longer operation times, higher complication rates or different surgical intents. Tissue samples were collected over a time span of 25 years. Importantly, quality criteria and surgical approaches of ovarian cancer surgery have significantly changed during this period. Although we included ‘residual disease status’ in our multivariate analysis to correct for this major criterion of ovarian cancer surgery, we were not able to properly control for the aforementioned parameters. Further, due to the retrospective and primarily histopathological character of our study, we were not able to correct for individual patient factors like lifestyle, fitness or comorbidities.

Though sample size certainly is a limitation of our study, the patient cohort studied herein has been extremely well characterized (first and second histopathological review by a specialized gynecological pathologist, broad range of clinical data including follow up available, IHC data available (*n* = 32)). Although the sample size of *n* = 143 might appear to be rather small, it is within the range of what several other pre-clinical, translational studies in ovarian cancer do report on [23,24,25]. We further analyzed serial sections of up to 94 lymph nodes per case. In total, we analyzed a number of 5042 lymph nodes in multiple serial sections. Since the primary aim of this study was to describe the phenomenon of ECG in ovarian cancer and its association with clinicopathological parameters, we think that *n* (ovarian cancer samples) = 143, *n* (lymph nodes analyzed) = 5042 is at least a sample size that might serve as a basis for further studies on this topic. Since IHC data were only available for 32 cases regression analysis might be statistically underpowered and needs to be interpreted with care. However, as GPER and Gal-3/8 (= positive prognosticators for overall survival) were positively associated with ICG and VU4H5 (= negative prognosticator for overall survival) was positively associated with ECG [12,13,14,15], regression analysis fits with biological rational of ICG/ECG and prognosis. 

Finally, whether the univariate prognostic significance of extracapsular spread may one day become of clinical relevance in ovarian cancer management and whether this may influence the decision on whether to perform lymphadenectomy or not, cannot be said until prospective, properly powered trials have been performed on this topic. 

## 4. Materials and Methods 

### 4.1. Patients

Tissue specimens obtained from 143 patients that had been diagnosed and treated with lymph node positive ovarian cancer were analyzed retrospectively. All patients had undergone radical ovarian cancer surgery at the Department of Obstetrics and Gynecology, Ludwig-Maximilians-University of Munich between 1990 and 2015. Surgery was performed by specialized gynecologic oncologists according to the national guidelines for ovarian cancer [8]. Patients received chemotherapy in either an adjuvant (*n* = 140) or a neo-adjuvant (*n* = 3) setting.

Histological characterization as well as histological tumor grading according to the WHO criteria were performed by a gynecological pathologist. Grading was binarized for statistical analysis. As long as not stated otherwise the term ‘low grade’ refers to low grade serous as well as to non-serous cancers graded as G1, and ‘high grade’ refers to high grade serous as well as non-serous cancers graded as G2 or G3. Staging was performed according to the FIGO criteria (8ed). Lymph node ratio (LNR) was calculated by the absolute number of resected lymph nodes divided by the number of metastatic lymph nodes. A lymph node ratio (LNR) of >0.3 was considered as high, ≤0.3 as low.

Clinical data were retrieved from patients’ charts and from the Munich Cancer Registry. Data were analyzed retrospectively. The outcome assessed was patients’ overall survival. 

### 4.2. Ethical Approval 

This study used tumor tissue that had initially been collected for histo-pathological diagnostics. At the time the tissue was examined for the current study all diagnostic procedures had already been fully completed and the tissue used was thus classified as left-over material. All patient data were fully anonymized, the Ethics Committee of the Ludwig-Maximilians-University (Munich, Germany) approved the study (227-09 and 18-392) and the study was performed according to the standards set in the declaration of Helsinki 1975. As per declaration of our ethics committee no written informed consent of the participants or permission to publish is needed given the circumstances described above. Researchers were blinded from patient data during experimental and statistical analysis. 

### 4.3. Assay Methods

#### 4.3.1. Determination of ECG and ICG

Tissue samples underwent routine histopathology processing. Briefly, specimens were fixed in neutral buffered formalin directly after resection and were submitted to fully automated paraffin embedding. FFPE tissue slides (thickness 2–5 µm) underwent semi-automated H&E staining. HE-stained tissue sections of the resected lymph nodes were evaluated on extracapsular lymph node involvement by two independent observers, including an experienced gynecological pathologist. ECG was defined as growth of tumor cells through or directly beyond the lymph node capsule invading into perinodal fat or into perinodal stroma [4]. Cases diagnosed for ECG in one or more than one lymph node were grouped into the ECG cohort. 

#### 4.3.2. Immunohistochemistry

Data regarding the protein-profile of the primary tumor were available in 32 cases and were derived from previous studies performed by our group [12,13,14,15,26,27,28,29]. Within the currents study staining data were re-analyzed with respect to the presence of either ECG or ICG of the corresponding lymph nodes. Mutation of TP53 was estimated by immunohistochemistry by using a score published earlier by our group and others [8,9].

#### 4.3.3. Statistical Analysis Methods

This study has been carried out according to the REMARK (Reporting Recommendations for Tumor Marker Prognostic Studies) criteria [30]. The IBM statistic package SPSS (version 24) was used to test data for statistical significance. Fisher’s exact test was performed to test categorical data for statistical independence. Student’s *t*-Tests was applied in the case of continuous variables. A logistic regression was run to ascertain the effects of four biomarkers on the likelihood of extracapsular spread. The Youden index (J = max_c_ {Sensitivity (c) + Specificity (c) − 1}) was calculated to illustrate the maximum potential effectiveness of this biomarker combination [31]. Survival analysis was done by applying the Log-Rank test and data are presented as Kaplan–Meier survival curves. Cox regression was performed on ECG and o-variables to test which parameter remains to be prognostic within multi-variate testing. Data were assumed to be statistically different in the case of *p* < 0.05. 

## 5. Conclusions

Our data demonstrate that extracapsular growth (ECG) of lymph node metastasis is a common feature of lymph node positive ovarian cancer. Since ECG was correlated with tumor grade, high lymph node ratio, and shortened overall survival, ECG may be interpreted as an indicator of tumor aggressiveness. Whether the pattern of lymph node involvement (extra- vs. intracapsular growth) may reproducibly add additional prognostic information in OC and whether this might aid to further individualize adjuvant therapy should be the subject of future studies.

## Figures and Tables

**Table 1:** Patients’ characteristics: Patients’ characteristics in cases diagnosed for intra-(ICG) and extracapsular (ECG) are displayed. Grading was binarized for statistical analysis. Low/high grade refers to the serous subtype only while G1/2/G3 represents grading of non-serous cases. *p*-values derive from Fisher’s exact test (in the case of categorical variables) and Student’s *t*-Test (in the case of continuous variables) are shown. ns: not significant.

Characteristic		ICG	ECG	*p*
		*n* or Median (Range)	*n* or Median (Range)	
**pT**	pT1/2	12	6	ns
	pT3	96	29	
**FIGO**	III	95	32	ns
	IV	13	3	
**Grade**	low or G1	18	1	0.043
	high or G2/3	88	34	
**Histology**	non-serous	6	8	0.006
	serous	102	27	
**Age**	(years)	59.62 (33.4–81.6)	59.46 (36.9–87.6)	ns
**Residual disease**	none	66	17	ns
	any	27	14	
**Total nodes resected**		38	37	ns
		(1–94)	(6–74)	
**Fraction positive nodes/total nodes resected**	≤0.3	79	11	<0.001
	>0.3	22	22	

**Table 2:** Univariate survival analysis: Clinicopathological parameters, lymph node ratio (LNR) and ECG/ICG and the combination of the latter two were tested for their prognostic significance. Significant results (*p* < 0.05) are indicated by bold font and *p*-values derived from relevant log rank tests are shown.

Characteristic	Univariate Analysis	
	Log Rank χ^2^ Test	*p*
FIGO (III vs. IV)	6.345	**0.012**
pT (pT1/2 vs. pT3)	0.743	0.389
Grade (G1 vs. G2/3)	9.862	**0.002**
Histology (other vs. serous)	0.425	0.515
Patient age (<60 years vs. ≥60 years)	2.960	0.085
Residual disease (none vs. any)	16.65	**<0.001**
Total nodes resected (<37 vs. ≥37)	0.223	0.637
LNR (≤0.3 vs. >0.3)	7.059	**0.008**
ECG vs. ICG	5.101	**0.024**
LNR > 0.3 and ECG vs. remaining	8.581	**0.003**

## Supplementary Materials

The following are available online at https://www.mdpi.com/2072-6694/11/7/924/s1, Table S1: Multivariate survival analysis.
