# Supplementary material for: Extracapsular Lymph Node Involvement in Ovarian Carcinoma
Source: Cancers (Basel). 2019 Jul 1;11(7):924. doi: 10.3390/cancers11070924 (PMC6678603; doi:10.3390/cancers11070924)
Supplement: Supplementary file 1 [file cancers-11-00924-s001.pdf]

cancers

Supplementary Materials: Extracapsular Lymph Node Involvement in Ovarian Carcinoma

Sabine Heublein, Heiko Schulz, Frederik Marmé, Martin Angele, Bastian Czogalla, Alexander Burges, Sven Mahner, Doris Mayr, Udo Jeschke and Elisa Schmoeckel

Table S1. Multivariate survival analysis.

Characteristic	Multivariate Analysis				Multivariate Analysis			
	HR	*p*	95% CI		HR	*p*	95% CI	
			Lower	Upper			Lower	Upper
pT (pT1/2 vs. pT3)	0.65	0.34	0.26	1.59	0.71	0.45	0.29	1.73
Grade (G1 vs. G2/3)	3.31	0.11	0.75	14.69	3.69	0.09	0.82	16.57
Histology (other vs. serous)	0.67	0.42	0.26	1.78	0.69	0.43	0.27	1.75
Patient age (< 60 years vs. ≥ 60 years)	1.32	0.33	0.75	2.3	1.31	0.34	0.75	2.28
Residual disease (none vs. any)	2.49	**0.01**	1.21	5.13	2.3	**0.02**	1.13	4.7
Total nodes resected (<37 vs. ≥37)	1.21	0.6	0.59	2.48	1.21	0.55	0.65	2.28
LNR (≤0.3 vs. >0.3)	1.14	0.7	0.58	2.27	n.i.	n.i.	n.i.	n.i.
ECG vs. ICG	1.25	0.47	0.68	2.29	n.i.	n.i.	n.i.	n.i.
LNR > 0.3 and ECG vs. remaining	n.i.	n.i.	n.i.	n.i.	1.76	0.08	0.94	3.28

Presence of residual tumor was the only variable that remained significant during multivariate testing. LNR and ECG/ICG failed to be of independent prognostic significance when tested as two separated single markers. However, their combination showed a trend of potentially acting as an independent negative prognosticator. Results of Cox Regression analysis are shown and significant results ($p < 0.05$) are indicated by bold font.

© 2019 by the authors. Licensee MDPI, Basel, Switzerland. This article is an open access article distributed under the terms and conditions of the Creative Commons Attribution (CC BY) license (http://creativecommons.org/licenses/by/4.0/).
